# Inhaled aerosolized insulin ameliorates hyperglycemia-induced inflammatory responses in the lungs in an experimental model of acute lung injury

**DOI:** 10.1186/cc12697

**Published:** 2013-04-28

**Authors:** Wei Fan, Koichi Nakazawa, Shinya Abe, Miori Inoue, Masanobu Kitagawa, Noriyuki Nagahara, Koshi Makita

**Affiliations:** 1 Department of Anesthesiology and Critical Care Medicine, Tokyo Medical and Dental University, 1-5-45 Yushima, Bunkyo-ku, Tokyo 113-8519, Japan; 2 Department of Comprehensive Pathology, Ageing and Developmental Sciences, Tokyo Medical and Dental University, 1-5-45 Yushima, Bunkyo-ku, Tokyo 113-8519, Japan; 3 Isotope Research Center, Nippon Medical School, 1-1-5 Sendagi, Bunkyo-ku, Tokyo, 113-8603 Japan

**Keywords:** aerosolized insulin, hyperglycemia, acute lung injury, inflammatory responses, lung, rabbit

## Abstract

**Introduction:**

Previous studies have shown that patients with diabetes mellitus appear to have a lower prevalence of acute lung injury. We assumed that insulin prescribed to patients with diabetes has an anti-inflammatory property and pulmonary administration of insulin might exert beneficial effects much more than intravenous administration.

**Methods:**

Twenty-eight mechanically ventilated rabbits underwent lung injury by saline lavage, and then the animals were allocated into a normoglycemia group (NG), a hyperglycemia group (HG), an HG treated with intravenous insulin (HG-VI) group or an HG treated with aerosolized insulin (HG-AI) group with continuous infusion of different fluid solutions and treatments: normal saline, 50% glucose, 50% glucose with intravenous insulin, or 50% glucose with inhaled aerosolized insulin, respectively. After four hours of treatment, the lungs and heart were excised *en bloc*, and then high-mobility group B1 concentration in bronchoalveolar lavage fluid, interleukin-8 and toll-like receptor 4 mRNA expression in bronchoalveolar lavage fluid cells, and lung myeloperoxidase activity were measured.

**Results:**

Treatment with both aerosolized insulin and intravenous insulin attenuated toll-like receptor 4 mRNA expressions in the bronchoalveolar lavage fluid cells. Interleukin-8 and toll-like receptor 4 mRNA expression was significantly lower in the HG-AI group than in the HG-IV group. The lung myeloperoxidase activity in the normal healthy group showed significantly lower levels compared to the NG group but not different compared to those of the HG, HG-VI and HG-AI groups.

**Conclusions:**

The results suggest that insulin attenuates inflammatory responses in the lungs augmented by hyperglycemia in acute lung injury and the insulin's efficacy may be better when administered by aerosol.

## Introduction

Sepsis or endotoxemia induces systemic inflammatory responses manifested by increased expression and release of proinflammatory cytokines, chemokines and adhesion molecules. These inflammatory responses activate inflammatory and structural cells, all of which release inflammatory mediators that elicit the typical pathophysiological changes of acute lung injury or acute respiratory distress syndrome (ALI/ARDS). It has been shown that hyperglycemia is associated with adverse outcomes, including the increased mortality of critically ill patients [1-7]. The increased mortality may be linked to the concurrent actions of hyperglycemia in modulating the systemic inflammatory process [8], increasing the risk of infection [8] and exaggerating coagulation [9]. Hyperglycemia enhances inflammatory responses accompanied by sepsis [10,11]. It is also known that hyperglycemia augments lung injury induced by lipopolysaccharide (LPS), as an intravenous glucose solution has been shown to increase serum high-mobility group B1 (HMGB1) levels and worsen pathophysiological findings in a rat model of LPS-induced lung injury [12]. In one *in vitro *study, hyperglycemia enhanced cytokine production in human peripheral blood mononuclear cells incubated with LPS [13].

Most investigations hitherto have focused on systemic inflammatory responses caused by sepsis or endotoxemia. The effects of hyperglycemia on established lung injury caused by direct insults have not been investigated. Contrary to the findings on the effects of hyperglycemia on sepsis or endotoxemia, clinical data indicate that diabetes confers protective effects against the development of ALI/ARDS [14]. In a large cohort study by Gong *et al. *[15], diabetes protected against the development of ARDS in patients at risk for ARDS in association with causes such as sepsis, trauma, massive transfusion and aspiration. In a prospective, multicenter study of patients with septic shock, glucose levels on admission were higher among patients who did not develop ALI/ARDS than among those who did [16,17]. Multiple reasons have been proposed to explain why diabetes may protect against ALI/ARDS, including the effect of hyperglycemia on the host response [14], but a recent cohort study concluded that diabetes was not associated with acute lung injury but was associated with cardiac overload [18]. Koh *et al*. also clarified that not diabetes but therapies associated with diabetes protected against adverse outcome [18]. According to one experimental study, diabetes therapies, such as insulin, can decrease the severity of lung injury by inhibiting the serum level of HMGB1 during the acute phase of LPS-induced lung injury [19]. Insulin treatment may exert beneficial metabolic effects beyond glucose control, as well as non-metabolic effects. The inhalation of aerosolized insulin is established as a rapid and safe route to reduce plasma glucose concentrations in diabetic rabbits [20]. In recent studies in humans, an inhaled dry powder formulation of recombinant regular human insulin has also shown favorable effects for diabetes [21,22]. Pulmonary administration of insulin may be beneficial for the treatment of lung injuries induced by direct insults if the patient is hyperglycemic.

The present study was conducted to investigate the effects of hyperglycemia on inflammatory responses in acute lung injury induced by whole lung lavage and to compare the effects of pulmonary or intravenous administration of insulin on ongoing inflammatory responses in the lungs. We assumed that if insulin has an anti-inflammatory property, pulmonary administration of insulin might exert beneficial effects much more than intravenous administration.

## Materials and methods

### Experimental protocol

All experimental protocols were reviewed and approved by the Animal Care and Use Committee of Tokyo Medical and Dental University and were performed according to the US National Institutes of Health guidelines. Thirty-one rabbits weighing between 3.1 and 3.3 kg were randomly assigned to five groups: normal, healthy control (NL group: *n *= 3), acute lung injury with normoglycemia (NG group: *n *= 7), acute lung injury with hyperglycemia (HG group: *n *= 7), acute lung injury with hyperglycemia treated with intravenous insulin (HG-VI group: *n *= 7) and acute lung injury with hyperglycemia treated with aerosolized insulin (HG-AI group: *n *= 7).

The animals were anaesthetized with intramuscular ketamine hydrochloride (20 mg/kg) and pentobarbital (15 mg/kg) and placed in a supine position. After subcutaneous infiltration with 1% lidocaine, a midline cervical incision was made, the animal was tracheostomized, and the trachea was cannulated by a tracheal tube (inner diameter, 3.5 mm). The normal healthy control animals were then sacrificed with pentobarbital, and the lungs and heart were excised *en bloc*. The lungs were treated by the same procedure as the four experimental groups, as described later. The other animals received mechanical ventilation with a Servo Ventilator 300 (Siemens-Elema AB, Solna, Sweden) under the following conditions: tidal volume, 10 ml/kg; respiratory rate, 25 breaths/minute; inspiratory:expiratory (I:E) ratio, 1:2; fraction of inspired oxygen (FiO_2_), 1.0; positive end-expiratory pressure (PEEP), 3 cmH_2_O. A 22-G venous catheter was introduced through the jugular vein for fluid and drug infusion. An arterial catheter was placed in the carotid artery to monitor arterial pressure and sample arterial blood. Lactated Ringer's solution was infused intravenously at a rate of 10 ml/kg/h throughout the study. Anesthesia was maintained using ketamine hydrochloride at 10 mg/kg/h and propofol at 10 mg/kg/h, and the animals were paralyzed with pancuronium at 0.1 mg/kg/h intravenously.

The arterial pressure was recorded on a polygraph system (RM6000; Nihon Kohden, Tokyo, Japan). Baseline measurements of lung mechanics and hemodynamics were performed after stabilization, and arterial blood was sampled for the analysis of blood glucose (Glucocard™ GT1670; Arkray Inc., Kyoto, Japan), partial pressure of oxygen in arterial blood (PaO_2_), partial pressure of carbon dioxide in the blood (PaCO_2_) and pH (ABL5; Radiometer Medical ApS, Copenhagen, Denmark). Once the baseline measurements were complete, lung lavage was performed with warm (38°C) normal saline (60 ml) to produce lung injury. The animals were disconnected from the ventilator and saline was instilled directly into the lungs via the tracheal tube. The animals were then ventilated under the previous settings for 15 s, and 10 ml of bronchoalveolar lavage fluid (BALF) was recovered for analysis of the HMGB1 levels and real-time polymerase chain reaction (RT-PCR) (baseline). Ventilation was then resumed for 90 s, and the rest of the saline was recovered by gentle suctioning. This lavage procedure was repeated every 10 minutes until the PaO_2_/FiO_2 _level was less than 150 mmHg. Control measurements were taken 60 minutes after confirming the establishment of lung injury, then the mode of ventilation was changed to low tidal volume with PEEP (tidal volume, 6 ml/kg; respiratory rate, 30 to 40 breaths/minute; I/E ratio, 1:2; FiO_2_, 1.0; PEEP, 10 cmH_2_O). The HG group, HG-VI group and HG-AI group then received a 50% glucose solution intravenously at an initial dose of 1.3 ml/kg over 30 minutes followed by 1.3 ml/kg/h, while the animals assigned to the NG group received an equivalent volume of normal saline. In the HG-VI group, a dose of insulin (23 IU/kg) (Humulin® R; Eli Lilly Japan, Kobe, Japan) was concomitantly administered intravenously at the infusion rate of 5.1 IU/kg/h. The HG-AI group received equivalent doses of 23 IU/kg of aerosolized insulin through an ultrasonic nebulizer (Misty™; ACOMA Medical Industry Co., Ltd., Tokyo, Japan) placed in the inspiratory limb of the ventilator circuit. The nebulizer chamber was primed with the study medication diluted in 5 ml normal saline. The diameter of the aerosol particle was 1 to 5 μm (published data from the company). Nebulization was accomplished in 30 minutes after the initiation of glucose infusion.

Arterial blood samples were obtained for blood glucose and blood gas analyses at 60, 120, 180 and 240 minutes after glucose or saline infusion. The arterial pressure, heart rat, and data on pulmonary mechanics (mean airway pressure [Pmean], plateau pressure [Pplateau], and minute volume [MV]) were also recorded at each time point.

Four hours after treatment, the animals were sacrificed by injection of a pentobarbital overdose. The lungs and heart were excised *en bloc*. BALF was harvested from the left lung with 25 ml of normal saline. The BALF and the fluid recovered at the induction of lung injury were centrifuged at 3,000 rpm (885 × g) for 15 minutes at 4°C. Cell-free supernatant was divided into several aliquots and stored at -80°C for measurement of HMGB1 levels. Cells were treated by TRIzol reagent (Invitrogen Co., Carlsbad, CA, USA) and stored at -80°C for measurement of mRNA.

### Measurement of BALF HMGB1

HMGB1 levels in BALF supernatant were measured using an enzyme linked immunosorbent assay (HMGB1 ELISA Kit II; Shino-Test Co., Sagamihara, Japan). HMGB1 was detected according to the manufacturer's protocols.

### mRNA analysis

Total RNA extracted from BALF cells using TRIzol reagent was treated with DNase to remove possible traces of contaminating DNA according to the manufacturer's instructions. cDNA was then synthesized from RNA by TaqMan^®^Reverse Transcription Reagents (Applied Biosystems, Foster, CA, USA) and quantitative RT-PCR was performed. PCR was performed with specific primers and TaqMan probes with FAST qPCR Master Mix Plus (Nippon Gene, Tokyo, Japan), and the PCR reaction was monitored with an ABI Prism 7900HT Sequence Detection System (Applied Biosystems). Relative mRNA expression was quantified using the 2-ΔΔCT method [23] with TaqMan Rabbit beta actin as internal control.

### Myeloperoxidase activity assay

The myeloperoxidase (MPO) activity was measured by a previously described method with modifications [24,25]. Homogenized lung tissues were collected in 1.5 ml microtube, mixed with 150 μl of 50 mM potassium phosphate buffer (pH 6.0) containing 0.5% hexadecyltrimethylammonium bromide and 5 mM ethylenediaminetetraacetic acid, incubated at 60°C for 2 hours, and centrifuged for 30 minutes at 14,000 rpm (19,279 × g) at 4°C. After 10 μl of the supernatant was added to 90 μl of 100 mM potassium phosphate buffer (pH 6.0) containing 0.167 mg/ml o-dianisidine hydrochloride and 0.0005% hydrogen peroxide, the change in absorbance at 460 nm (e460 = 11.3/mM/cm) [26] was followed for three-minute periods at regular intervals (no less than 10 seconds) by a spectrophotometer (Gene Spec V; Hitachi High-Tech Manufacturing & Service Co., Ltd., Tokyo, Japan). The total protein concentration was measured with a Coomassie (Bradford) Protein Assay Kit (Thermo Fisher Scientific, Rockford, IL, USA) with bovine serum albumin (Thermo Fisher Scientific) according to the manufacturer's protocols. The MPO specific activity (mU/mg protein) was calculated. One unit of MPO activity was defined as that required to degrade 1 μmol of H_2_O_2 _per minute at 25°C.

### Histopathologic examination

The right upper lobe of the lung was inflation-fixed with formaldehyde solution through the right main bronchus at 20 cmH_2_O. For at least 48 h after fixation, the lung was embedded in paraffin. Next, 4-μm-thick sections were stained with hematoxylin and eosin (HE) and examined under a light microscope. Three observers blinded to the nature of the experiment scored lung injury from 0 (no damage) to 3 (maximal damage) according to three assessment categories: edema, alveolar congestion and infiltration of polymorphonuclear neutrophils (PMN) in the airspace or vessel walls. Edema and alveolar congestion were defined as the presence of intraalveolar pink staining fluid and the presence of red blood cells in the alveolar space, respectively.

### Wet-to-dry weight ratio of the lung

Pulmonary edema was also assessed using a wet-to-dry weight (W/D) ratio. The right lower lobe of the lung was weighed and placed into a desiccator (Isuzu Seisakusho Co., LTD, Niigata, Japan) for one week for analysis of the W/D ratio.

### Statistical analysis

Data values are expressed as means ± SD or medians and interquartile ranges, as appropriate. All statistical analyses of recorded data were performed using the Excel statistical software package (Ekuseru-Toukei 2010; Social Survey Research Information Co., Ltd., Tokyo, Japan). Comparisons between before injury and after injury were made by Wilcoxon signed-rank test for HMGB1 concentration and PCR. MPO activity of each treatment group was compared with that of a NL group using the Kruskal-Wallis test, followed by the Steel's multiple comparisons. The other data were analyzed by the Kruskal-Wallis test, followed by a Steel-Dwass test for multiple comparisons. Values of *P *less than 0.05 were considered statistically significant.

## Results

### Blood gases and pulmonary mechanics

PaO_2 _decreased after induction of lung injury, but the values were elevated after application of 10 cmH_2_O PEEP. The PaO_2 _values of the HG group were significantly lower at the end of the experiment compared to those of the HG-VI and HG-AI groups (*P *= 0.0211, *P *= 0.0095, respectively) (Table 1). There were no significant differences in Pmean, Pplateau or MV values among the groups during the experiment.

**Table 1:** Arterial blood gas and hemodynamic data

		Baseline	After Injury	One hour after treatment	End of experiment
pH	NG	7.38 ± 0.04	7.35 ± 0.06	7.27 ± 0.04	7.29 ± 0.03
	HG	7.39 ± 0.04	7.34 ± 0.05	7.28 ± 0.05	7.26 ± 0.05
	HG-VI	7.39 ± 0.04	7.37 ± 0.06	7.30 ± 0.03	7.32 ± 0.02
	HG-AI	7.39 ± 0.02	7.33 ± 0.06	7.34 ± 0.05	7.34 ± 0.05
PaCO_2 _(mmHg)	NG	40 ± 6	46 ± 12	57 ± 7	58 ± 3
	HG	39 ± 4	49 ± 11	60 ± 5	64 ± 11
	HG-VI	39 ± 4	45 ± 11	47 ± 5	47 ± 6
	HG-AI	38 ± 3	48 ± 5	50 ± 8	53 ± 7
PO_2_/FiO_2_	NG	620 ± 36	105 ± 25	503 ± 71**	565 ± 62**
	HG	617 ± 16	74 ± 18	420 ± 124**	378 ± 118**
	HG-VI	616 ± 28	77 ± 19	586 ± 68**	609 ± 55** ^#^
	HG-AI	637 ± 21	80 ± 21	588 ± 53**	637 ± 13** ^##^
HR (beats/minute)	NG	236 ± 34	260 ± 44	254 ± 36	261 ± 42
	HG	259 ± 39	271 ± 34	269 ± 38	253 ± 43
	HG-VI	232 ± 49	249 ± 33	259 ± 24	255 ± 22
	HG-AI	239 ± 21	256 ± 15	270 ± 20	258 ± 32
MAP (mmHg)	NG	90 ± 17	106 ± 28	99 ± 23	88 ± 25
	HG	85 ± 13	106 ± 24	95 ± 20	109 ± 25
	HG-VI	85 ± 7	93 ± 15	71 ± 13	71 ± 18
	HG-AI	90 ± 8	102 ± 7	90 ± 10	103 ± 13

NG, acute lung injury with normoglycemia group; HG, acute lung injury with hyperglycemia group; HG-VI, acute lung injury with hyperglycemia treated with intravenous insulin group; HG-AI, acute lung injury with hyperglycemia treated with aerosolized insulin group; HR, heart rate; MAP, mean arterial pressure. All values are mean ± SD. **P <0.01 compared with after injury; ^##^*P *<0.01, ^#^*P *<0.05 compared with the HG group.

### Blood glucose

**Figure 1:**
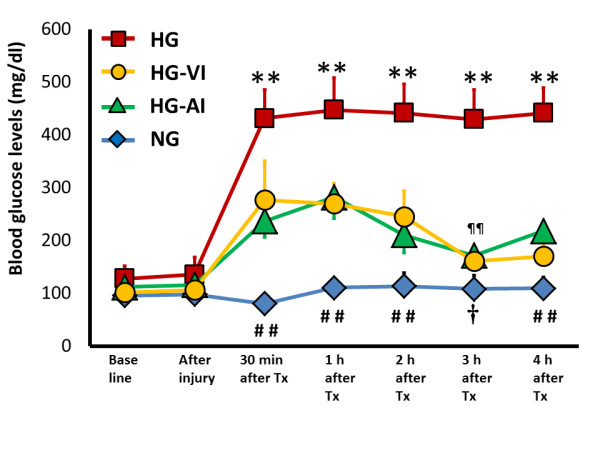
**Changes in blood glucose levels during the experiment**. NG, acute lung injury with normoglycemia group; HG, acute lung injury with hyperglycemia group; HG-VI, acute lung injury with hyperglycemia treated with intravenous insulin group; HG-AI, acute lung injury with hyperglycemia treated with aerosolized insulin group; Tx, treatment. Data are mean ± SD. **P <0.01 compared with each time-point in other groups; ^##^*P *<0.01 compared with each time-point in the HG-VI group and HG-AI group; ^†^*P *<0.05 compared with the HG-VI group; ^¶¶^*P *<0.01, HG-AI group compared with the NG group.

Blood glucose levels in the HG group ranged between 430 and 448 mg/dl during the experiment, whereas those in the NG group showed normal levels (81 to 114 mg/dl). The blood glucose levels in the HG-VI and HG-AI group were significantly lower than those in the HG group, but they were not reduced to normal levels. The lowest blood glucose level in the HG-VI group (162 ± 28 mg/dl)/HG-AI group (172 ± 17 mg/dl) was observed at 180 minutes after treatment (Figure 1).

### BALF analysis

**Figure 2:**
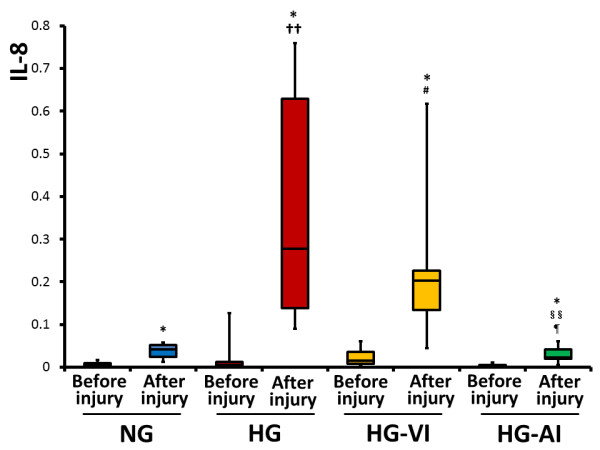
**mRNA expression of Interleukin-8 in bronchoalveolar lavage fluid cells before injury and after injury**. NG, acute lung injury with normoglycemia group; HG, acute lung injury with hyperglycemia group; HG-VI, acute lung injury with hyperglycemia treated with intravenous insulin group; HG-AI, acute lung injury with hyperglycemia treated with aerosolized insulin group; IL-8, interleukin-8. Boxes extend from the 25^th ^to 75^th ^percentile; the horizontal line shows the median. Error bars show the minimum and maximum. *P <0.05 compared with before injury; ^††^*P *<0.01 ^#^*P *<0.05 compared with after injury in NG; ^§§^*P *<0.01 compared with after injury in HG group; ^¶^*P *<0.05 compared with after injury in HG-VI group.

Gene expressions of interleukin-8 (IL-8) in the BALF cells rose significantly at the end of the experiment in all groups (before lung injury vs. end of the experiment: NG, *P *= 0.0180; HG, *P *= 0.0180; HG-VI, *P *= 0.0180; HG-AI, *P *= 0.0180 respectively). The gene expression of IL-8 was lower in the HG-AI group than in the HG and HG-VI groups (HG-AI vs. HG, *P *= 0.0095; HG-AI vs. HG-VI, *P *= 0.0211), but there was no significant difference between the HG-AI and NG groups (*P *=0.8960) (Figure 2).

**Figure 3:**
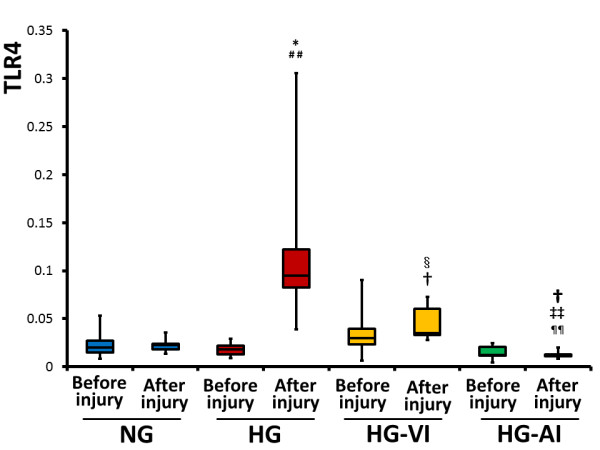
**mRNA expression of Toll-like receptor 4 in bronchoalveolar lavage fluid before injury and after injury**. NG, acute lung injury with normoglycemia group; HG, acute lung injury with hyperglycemia group; HG-VI, acute lung injury with hyperglycemia treated with intravenous insulin group; HG-AI, acute lung injury with hyperglycemia treated with aerosolized insulin group; TLR4, Toll-like receptor 4. Boxes extend from the 25^th ^to 75^th ^percentile; the horizontal line shows the median. Error bars show the minimum and maximum. *P <0.05 compared before injury in the HG group; ^##^*P *<0.01 compared with after injury in the NG group; ^§^*P *<0.05 compared with after injury in the HG group; ^†^*P *<0.05 compared with after injury in the NG group; ^‡‡^*P *<0.01 compared with after injury in the HG group; ^¶¶^*P *<0.01 compared with after injury in the HG-VI group.

The gene expressions of toll-like receptor 4 (TLR4) did not differ before and after the experiment in the NG (*P *= 1.0), HG-VI (*P *= 0.3105) and HG-AI (*P *= 0.6121) groups, but those in the HG group (*P *= 0.0180) were significantly enhanced after the experiment. The TLR4 expressions were significantly lower in the HG-AI group than in the other groups at the end of the experiment (*P *= 0.0437 vs. NG, *P *= 0.0095 vs. HG, *P *= 0.0095 vs. HG-VI, respectively) (Figure 3).

**Figure 4:**
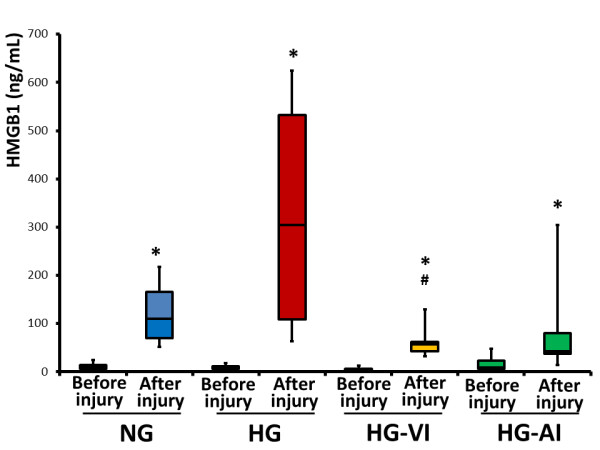
**Bronchoalveolar lavage fluid high-mobility group B1 levels before injury and after injury**. NG, acute lung injury with normoglycemia group; HG, acute lung injury with hyperglycemia group; HG-VI, acute lung injury with hyperglycemia treated with intravenous insulin group; HG-AI, acute lung injury with hyperglycemia treated with aerosolized insulin group; HMGB1, high-mobility group B 1. Boxes extend from the 25^th ^to 75^th ^percentile; the horizontal line shows the median. Error bars show the minimum and maximum. *P <0.05 compared before injury; ^#^*P *<0.05 compared with after injury in the HG group.

The BALF HMGB-1 levels increased at the end of the experiment in the NG (*P *= 0.0180), HG (*P *= 0.0180), HG-VI (*P *= 0.0180) and HG-AI (*P *= 0.0180) groups. The BALF HMGB1 levels at the end of the experiment of the HG-VI group are significantly lower than those of the HG group (*P *= 0.0306) (Figure 4).

### Myeloperoxidase activity assay

**Figure 5:**
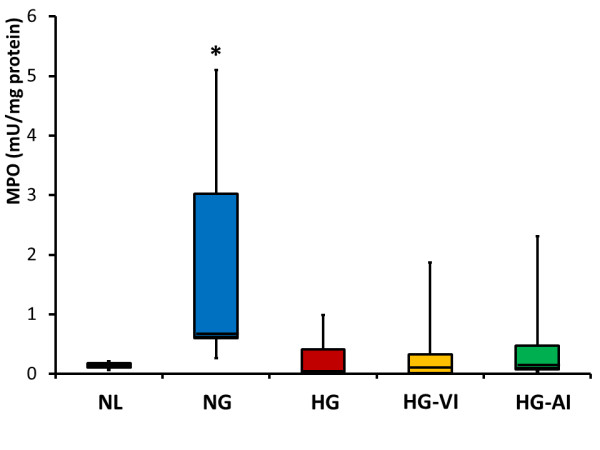
**Lung tissue myeloperoxidase specific activity**. mU/mg protein means milliunit per mg protein of lung tissue. NL, normal healthy control group; NG, acute lung injury with normoglycemia group; HG, acute lung injury with hyperglycemia group; HG-VI, acute lung injury with hyperglycemia treated with intravenous insulin group; HG-AI, acute lung injury with hyperglycemia treated with aerosolized insulin group; MPO, myeloperoxidase. Boxes extend from the 25^th ^to 75^th ^percentile; the horizontal line shows the median. Error bars show the minimum and maximum. *P <0.05 compared with the NL group.

The MPO activity of the lung in the NG group was significantly higher than those in the NL groups (*P *= 0.0240), but no significant differences were found between the NL and the other groups (Figure 5).

### Lung pathology

**Figure 6:**
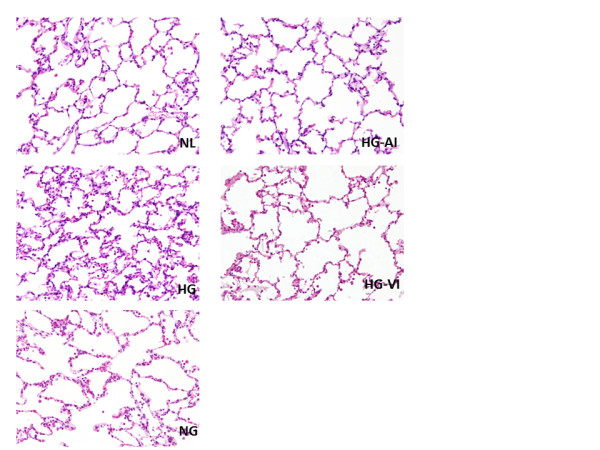
**Representative micrograph of histopathology in each experimental group (HE stain ×400)**. NL, normal healthy control group; NG, acute lung injury with normoglycemia group; HG, acute lung injury with hyperglycemia group; HG-VI, acute lung injury with hyperglycemia treated with intravenous insulin group; HG-AI, acute lung injury with hyperglycemia treated with aerosolized insulin group. Alveolar congestion, edema and infiltration of polymorphonuclear neutrophils were more prominent in the HG group than in other groups. Alveolar congestion, edema had lower scores in the HG-AI group compared with the HG-VI group. Those parameters showed no difference between the NG group and the HG-AI group.

Representative microscopic images are shown in Figure 6. Lung injury was more prominent in the HG group than in the NG, HG-VI and HG-AI groups, when judged on the basis of the scores for edema, alveolar congestion and infiltration of PMN. No difference in the degree of injury was found between the HG-AI group and NG group, but the degrees of edema and alveolar congestion of HG-AI group were significantly lower than those in the HG-VI group (Table 2).

**Table 2:** Histology injury score and lung wet-to-dry ratio at the end of the experiment

	NL	NG	HG	HG-VI	HG-AI
Alveolar congestion	0.11 ± 0.33	^#^ 0.67 ± 0.48	^## ^** 2.48 ± 0.51	^## ^** ^§§^ 1.71 ± 0.56	^## §§ ¶¶^ 0.90 ± 0.30
Edema	0 ± 0	0.19 ± 0.4	^## ^** 2.24 ± 0.70	^## ^** ^§§^ 1.67 ± 0.80	^§§ ¶¶^ 0 ± 0
Infiltration/ aggregation of neutrophils	0 ± 0	^##^ 0.95 ± 0.59	^## ^** 2.33 ± 0.73	^## §§^ 1.33 ± 0.66	^## §§^ 1.05 ± 0.59
Wet-to-dry ratio	NA	7.99 ± 1.16	8.37 ± 1.52	8.69 ± 2.07	* ^§^ 6.33 ± 1.00

NL, normal healthy control group; NG, acute lung injury with normoglycemia group; HG, acute lung injury with hyperglycemia group; HG-VI, acute lung injury with hyperglycemia treated with intravenous insulin group; HG-AI, acute lung injury with hyperglycemia treated with aerosolized insulin group; NA, not available. Values shown are mean ± SD. ^##^*P *<0.01, ^#^*P *<0.05 compared with the NL group; **P <0.01, *P <0.05 compared with the NG group; ^§§^*P *<0.01, ^§^*P *<0.05 compared with the HG group; ^¶¶^*P *<0.01 compared with the HG-VI group.

### Wet-to-dry weight ratios

The W/D weight ratios were significantly lower in the HG-AI group than in the HG (*P *= 0.0241) and NG groups (*P *= 0.0172). Those of the NG, HG and HG-VI groups were not significantly different from each other (Table 2).

## Discussion

Several studies have investigated the influences of hyperglycemia on inflammatory response in lungs injured by indirect insults [19,27]. We found that hyperglycemia enhanced inflammatory responses in the acutely injured lung and that inhaled insulin ameliorated these responses, as shown in reduction of IL-8 and TLR4 mRNA expressions in the BALF cells, even greater than those treated by intravenous insulin. This suggested the preferential effects of insulin in reducing the levels of these cytokines and insulin's apparent anti-inflammatory role in counterbalancing the physiologic responses to high glucose [28]. Recently, intravenous insulin treatment showed inhibition on the expression of nuclear factor-kappa B (NF-κB) and TLR4 in a LPS-induced lung injury model [29], but the present results have just confirmed an inference that insulin in an inhaled form capable of reaching the alveoli may exert a local anti-inflammatory effect [30].

The animals in the present study were treated with lung protective ventilation, a gold standard therapy in the respiratory management of ALI/ARDS. Ventilation strategies have been known to modulate inflammatory responses in both normal and injured lungs [31]. Our group has investigated the effects of PEEP on the intrapulmonary inflammatory responses induced by whole lung lavage using rabbits. PEEP above the lower inflection point on the pressure volume curve decreased IL-8 levels in BALF and serum from rabbits subjected to lung injury by whole lung lavage [32]. In a later experiment with the same lung injury model, low tidal volume with 10 cmH_2_O PEEP or airway pressure released ventilation significantly reduced the HMGB1 levels in BALF compared to conventional tidal volume with low PEEP [33]. Contrary to our expectations, the expression of TLR4 was concealed even after lung injury in our NG group. We can think of two mechanisms that may explain this concealment of TLR4. First, ventilator associated lung injury was minimized in the present study through the use of a low tidal volume with 10 cmH_2_O PEEP. Given the key role of TLR4 in both ventilator induced lung injury [34] and bacterial infection or sepsis [35], we speculate that the lung protective ventilation might have suppressed TLR4 mRNA expression in our NG group. Second, hyperglycemia in itself induces the expression of TLR4 mRNA. An *in vitro *experiment showed that high glucose (270 mg/dl) induced enhanced TLR4 expression in cultured human monocytes after six hours of treatment [36]. TLR4 initiates signaling through intracellular pathways that lead to activation of transcription factors, such as NF-κB, which in turn results in the transcription of proinflammatory cytokine genes [35]. These findings indicate that hyperglycemia is associated with up-regulation of TLR4 expression and subsequent proinflammatory cytokine expression, such as IL-8.

Even though hyperglycemia promoted the mRNA expression of IL-8 or TLR4 and PMN aggregation, it diminished the MPO activity in the lung tissue. MPO is a hemoprotein abundantly expressed in PMN and is secreted during PMN activation. MPO plays an important role in neutrophil bactericidal action by catalyzing chloride ion oxidation to hypochlorous acid, which can be a potent antimicrobial agent [37]. Recent evidence suggests that hypochlorous acid can also induce host cell damage, particularly under inflammatory conditions, and thereby contribute to the development of a number of diseases, including acute lung injury [38]. The levels of MPO activity per lung tissue protein of the HG, HG-VI and HG-AI groups were suppressed to the level of the NL group regardless of use of insulin in the present study. This suggests that insulin fails to restore MPO activity once a hyperglycemic state has been established. Yet as the histopathology shows, the degree of PMN infiltration was much higher in the HG group than in the other groups. We could not clarify how MPO activity depression itself affects or modifies the lung injury in the present model. Longer term studies are necessary to discriminate immune-compromised effects from anti-inflammatory effects.

Besides the fact that insulin down-regulates TLR4 expression that can end in an anti-inflammatory effect, it is also known that glycogen synthase kinase (GSK)-3, which is a key regulatory switch for the phosphatidylinositol 3-kinase (PI3K)/protein kinase B (Akt) signaling pathway, is also modulated by insulin [39,40]. GSK-3 promotes expression of a subset of genes of inflammatory molecules activated by NF-κB [41], while GSK-3 inhibition provides protection from inflammatory conditions [42]. Insulin binds to the insulin receptor, which, in turn, activates the PI3K pathway and this indirectly activates Akt via phosphorylation. Akt then phosphorylates and inactivates several target proteins, including GSK-3 [43]. Kidd *et al*. reported that a low dose of insulin (0.1 IU/kg), which did not affect blood glucose levels, inhibited inflammation during endotoxemia by activation of the PI3K/Akt pathway [44].

It is also suggested that insulin may have another favorable effect on lung injury. Insulin has been proved to decrease edema formation by inducing Na/K-ATPase translocation [45,46], and reduce accumulation of leukocytes in inflammatory lesions [47]. Simultaneously, similar to these results, our experiment also demonstrated that the histopathologic changes and the W/D ratio were reduced in the HG-AI group. These results may provide a possible mechanism that explains the anti-inflammatory activity of aerosolized insulin.

Because frequent blood glucose measurement was required, we used a compact device for self**-**monitoring in humans. This device can register values as high as 600 mg/dl, and accuracy seems acceptable unless applied for diagnostic purposes, such as the glucose tolerance test. We administered a glucose dose necessary to keep the blood glucose level above 400 mg/dl. This target concentration of blood glucose seems somewhat high, but it is a concentration encountered in critically ill patients. The same blood glucose levels have been maintained in earlier studies exploring the effects of hyperglycemia on inflammatory responses associated with endotoxemia [48-50]. It should be remembered that hyperglycemia induced by high dose glucose infusion may differ from hyperglycemia due to insulin resistance frequently seen in critically ill patients. Therefore, the results of the present study should be cautiously interpreted in patients with hyperglycemia due to insulin resistance. However, induction of mechanical ventilation and acute lung injury might predispose patients to stress responses, which impaired insulin sensitivity. Inflammation is known to impair insulin sensitivity in part via the activation of the TLR4 [51]. The dose of aerosolized insulin chosen in the current experiment, which was required to decrease blood glucose, was difficult to determine, but we performed a preliminary experiment to measure dose response curves for aerosolized insulin from 50 IU to 80 IU to obtain blood glucose level below 200 mg/dl. We found that the minimum required dose was 70 IU. Because the weight range of the animals was between 3.1 and 3.3 kg, we administered 23 IU/kg of aerosolized insulin. In the HG-IV group, an equivalent dose of insulin was administered by continuous intravenous infusion during the experimental course (4.5 h). Although the dose was not enough to normalize the blood glucose levels, it was enough to ameliorate local inflammatory responses.

The hyperglycemia-induced production of proinflammatory cytokines may be partly explained by the mechanisms of hyperglycemia-induced hyperosmosis. Booth *et al. *[52] demonstrated that intraperitoneal injection of 25 mmol/l D-glucose significantly increased leukocyte rolling and adherence in the mesenteric venules and leukocyte transmigration compared with control rats injected with Krebs-Henseleit solution. This response, however, was not elicited by the same concentration of L-glucose, an enantiomer of D-glucose. Hyperosmosis in itself does not appear to be a key exaggeration of acute inflammatory responses in the lungs.

As is often the problem with experiments using rabbits, the ELISA kits for measurement of most pro- and anti-inflammatory cytokines are not commercially available at present. The increased expression of IL-8 or TLR4 mRNA might not reflect an increased release of inflammatory mediators and vice versa. mRNA expression might be sometimes useful, but sometimes far from perfect, in predicting protein expression levels. It is known that the degree of correlation between gene expression of and protein levels varied among different cytokines [53]. Also each protein has a very different half-life as the result of varied protein synthesis and degradation [54]. Further study is necessary to examine if the mRNA expression levels are correlated with protein production and protein degradation. Nonetheless, our previous study indicated that both BALF and serum IL-8 protein levels were significantly elevated in the same lung injury model and those were reduced by lung protective strategy [32]. We, therefore, speculate that the different levels of mRNA expression of IL-8 among the groups reflect different concentrations in BALF IL-8 protein. The more prominent neutrophil infiltration in the lungs in hyperglycemia may be explained by the higher levels of IL-8 in the lungs.

## Conclusions

In summary, the results suggest that aerosolized insulin alleviates inflammatory responses augmented by hyperglycemia in acute lung injury.

## Key messages

• Hyperglycemia augmented inflammatory responses in the lungs in acute lung injury caused by lung lavage.

• Hyperglycemia causes greater PMN infiltration but low MPO activities in the lung tissue.

• Inhalation of aerosolized insulin alleviates inflammatory responses much more than does intravenous administration.

• Aerosolized insulin shows effects beyond mitigating hyperglycemia.

## Abbreviations

Akt: protein kinase B; ALI: acute lung injury; ARDS: acute respiratory distress syndrome; BALF: bronchoalveolar lavage fluid; GSK-3: glycogen synthase kinase-3; HE: hematoxylin and eosin staining; HG: acute lung injury with hyperglycemia group; HG-AI: acute lung injury with hyperglycemia treated with aerosolized insulin; HG-VI: acute lung injury with hyperglycemia treated with intravenous insulin; HMGB1: high-mobility group B1; I:E: inspiratory:expiratory ratio; IL-8: interleukin-8; LPS: lipopolysaccharide; MPO: myeloperoxidase; MV: minute volume; NG: acute lung injury with normoglycemia group; NL: normal healthy control group; PaCO_2_: partial pressure of carbon dioxide in the blood; PaO_2_: partial pressure of oxygen in arterial blood; PEEP: positive end-expiratory pressure; PI3K: phosphatidylinositol 3-kinase; Pmean: mean airway pressure; PMN: polymorphonuclear neutrophils; Pplateau: plateau pressure; RT-PCR: real-time polymerase chain reaction; TLR4: toll-like receptor 4; W/D: wet-to-dry weight ratio

## Competing interests

The authors declare that they have no competing interests.

## Authors' contributions

WF and KN designed and carried out the experiment, the acquisition of data, analysis and interpretation of data, and wrote the manuscript. SA, MI and MK took partial responsibility for RT-PCR and histopathologic examination. NN participated in and conducted the myeloperoxidase activity assay. KM made substantial contributions to conception and design of the study, and to the draft of the manuscript. All authors read and approved the final manuscript.
